# First APN-led Palliative Psychiatry Initiative The aging population has led to a rising number of multimorbid psychiatric inpatients, often receiving fragmented care with limited palliative approaches

**DOI:** 10.1192/j.eurpsy.2026.10671

**Published:** 2026-07-31

**Authors:** G. Gübel

**Affiliations:** Psychiatrische Universitätsklinik Zürich (PUK), 8032 Zürich, Switzerland

## Abstract

**Introduction:**

The aging population has led to a rising number of multimorbid psychiatric inpatients, often receiving fragmented care with limited palliative approaches.

**Objectives:**

This project aims to integrate palliative care concepts into psychiatric inpatient settings, emphasizing person-centered, ethical, and coordinated end-of-life care.

**Methods:**

The initiative is structured in three phases: (1) a scoping review to identify existing palliative care models applicable to geriatric psychiatry; (2) concept development guided by the Theory of Change framework, and (3) a pilot intervention involving staff training, standard operating procedures, and mixed-method evaluation. The ultimate goal is to develop an adaptable palliative care model for psychiatric settings.

**Results:**

Preliminary outcomes include clearer treatment goals, improved ethical decision-making, and enhanced support for vulnerable patients and staff. This work lays the foundation for a national network and model replication.

**Image 1:**
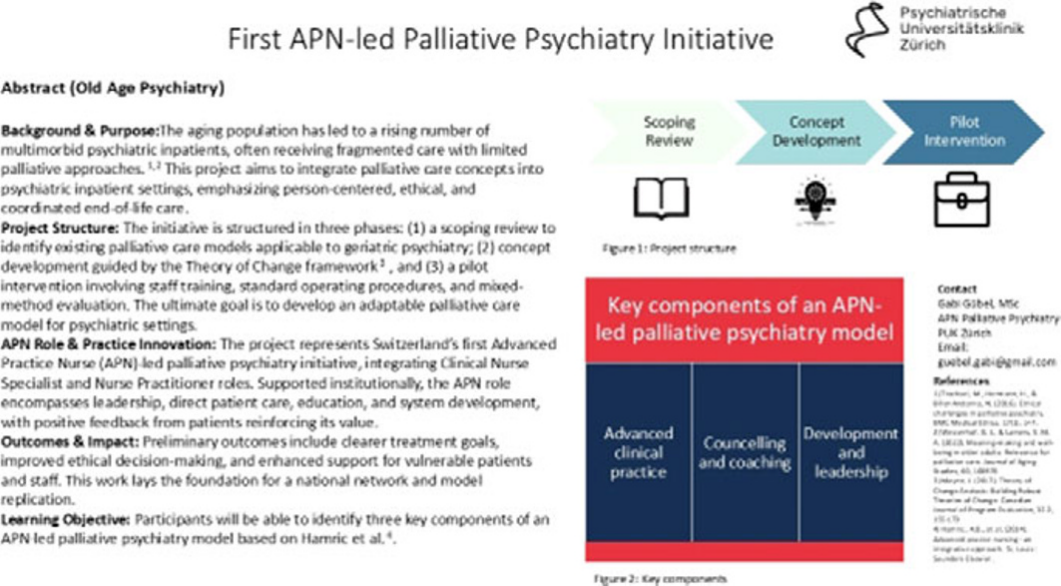
Image 1: Long description.

**Conclusions:**

Preliminary outcomes include clearer treatment goals, improved ethical decision-making, and enhanced support for vulnerable patients and staff. This work lays the foundation for a national network and model replication.

**Disclosure of Interest:**

None Declared

## Long descriptions

### Image 1: Long description

The poster is titled First A P N led Palliative Psychiatry Initiative.

On the left, an Abstract for Old Age Psychiatry contains five sections.

1. Background and Purpose. Discusses the rising number of multimorbid psychiatric inpatients and the goal to integrate person-centered palliative care.

2. Project Structure. Outlines three phases: scoping review, concept development, and pilot intervention.

3. A P N Role and Practice Innovation. Describes the first Advanced Practice Nurse led initiative in Switzerland, focusing on leadership, patient care, and education.

4. Outcomes and Impact. Notes improved treatment goals and ethical decision-making.

5. Learning Objective. Participants will identify three key components of the model.

On the top right, a horizontal flowchart labeled Figure 1: Project structure shows three chevron-shaped arrows pointing right.

- Phase 1: Scoping Review, represented by an open book icon.

- Phase 2: Concept Development, represented by a lightbulb icon.

- Phase 3: Pilot Intervention, represented by a medical bag icon.

Below the flowchart is Figure 2: Key components of an A P N led palliative psychiatry model. This is a rectangular diagram with a red header and a dark blue body divided into three vertical columns.

- Left column: Advanced clinical practice.

- Middle column: Counselling and coaching.

- Right column: Development and leadership.

The bottom right corner contains contact information for Gabi Gübel, M S c, and a list of four academic references.

Navigate back to Image 1:.

